# Supplementary material for: Orphan Nuclear Receptor Nur77 Mediates the Lethal Endoplasmic Reticulum Stress and Therapeutic Efficacy of Cryptomeridiol in Hepatocellular Carcinoma
Source: Cells. 2022 Dec 1;11(23):3870. doi: 10.3390/cells11233870 (PMC9737475; doi:10.3390/cells11233870)
Supplement: Supplementary file 1 [file cells-11-03870-s001.zip › cells-1996545-supplementary.pdf]

Supplementary Information

Orphan Nuclear Receptor Nur77 Mediates the Lethal Endoplasmic Reticulum Stress and Therapeutic Efficacy of Cryptomeridiol in Hepatocellular Carcinoma

Xudan Li [1,†], Quancheng Chen [1,†], Jie Liu [1], Shenjin Lai [1], Minda Zhang [1], Tidong Zhen [1], Hongyu Hu [2], Xiang Gao [1], Alice S. T. Wong [3] and Jin-Zhang Zeng [1,*]

[1] State Key Laboratory of Cellular Stress Biology, Fujian Provincial Key Laboratory of Innovative Drug Target Research, School of Pharmaceutical Sciences, Faculty of Medicine and Life Sciences, Xiamen University, Xiamen 361102, China
[2] Xingzhi College, Zhejiang Normal University, Lanxi 321004, China
[3] School of Biological Sciences, University of Hong Kong, Pokfulam Road, Hong Kong, China
* Correspondence: jzzeng@xmu.edu.cn; Tel.: +86-0592-2187221
† These authors contributed equally to this work.

Supplementary Figure S1

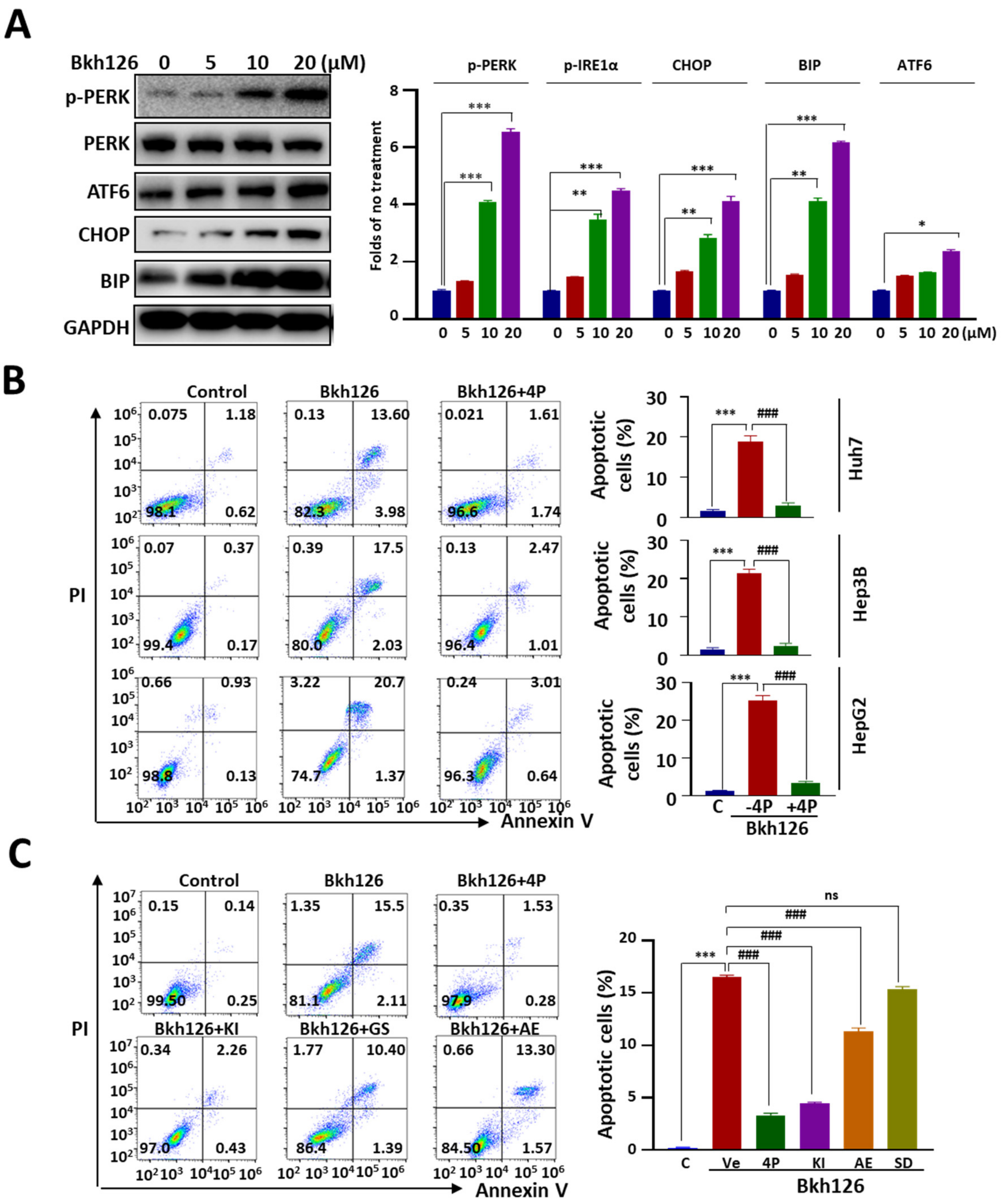

Supplementary Figure S1. (A) HepG2 cells were treated with different concentrations of Bkh126 for 24 h. The expression and phosphorylation of PERK and ATF6, CHOP, BIP were blotted. **(B)** HepG2, Huh-7 and Hep3B were pre-treated with 500 μM 4-PBA (ER stress inhibitor) and then 20 μM Bkh126 for 24 h. Flow cytometry was used to assay the apoptotic rates through Annexin V/PI staining and quantification. **(C)** HepG2 cells were pre-treated with 500 μM 4-PBA (ER stress inhibitor), 5 μM KRIA6(IRE-1ainhibitor), 300 μM AEBSF (ATF6 inhibitor), 2 μM GSK2606414 (PERK inhibitor) and then 20 μM Bkh126 for 24 h. Flow cytometry was used to assay the apoptotic rates through Annexin V/PI staining and quantification. **p*<0.05, ***p*<0.01, ****p*<0.001, ###*p*<0.001, ns, not significant.

Supplementary Figure S2

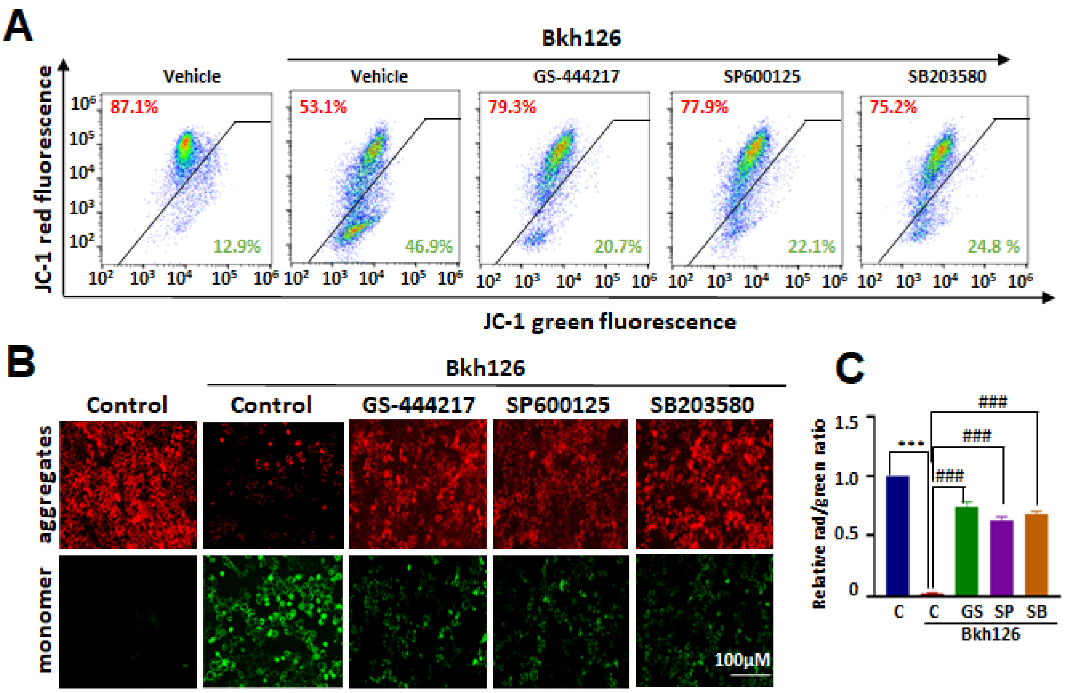

Supplementary Figure S2. HepG2 cells were treated with vehicle or 20 μM Bkh126 in the presence of either GSK-444217 (ASK1 inhibitor), or SP600125 (JNK inhibitor), or SB203580 (p38 inhibitor) for 24 h. The cells were then subjected to imaging by flow cytometry **(A)**, fluorescent microscope **(B)** and quantification **(C)**. ***p<0.001, ###p<0.001.

Supplementary Figure S3

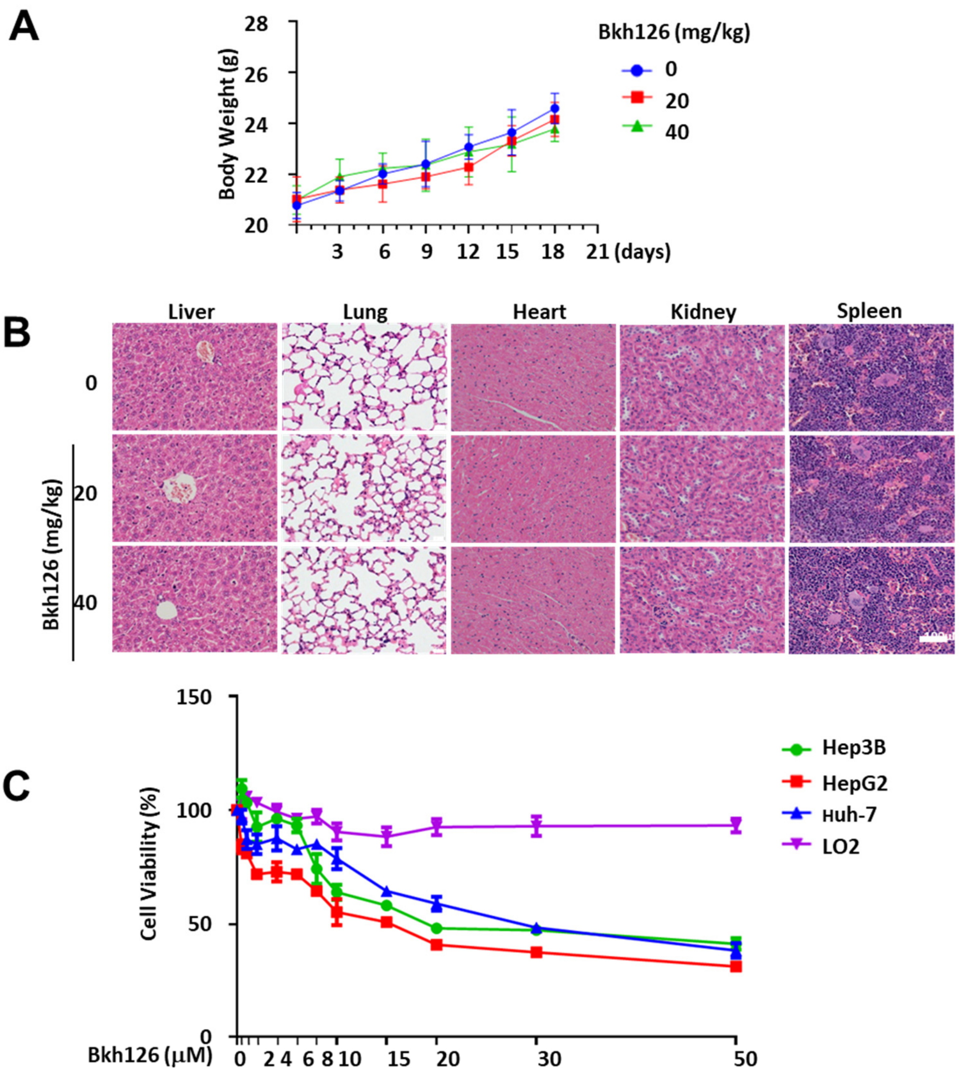

D

Bkh126 inhibits HCC cell growth (MTT)

Cell lines	IC_{50} (μM)
Hep3B	25.72 ± 1.3
HepG2	17.36 ± 0.9
Huh77	33.14 ± 1.1
LO2	> 50

Supplementary Figure S3. The male BALB/c nude mice harbored HepG2 xenograft tumors were treated with vehicle or Bkh126 (20 or 40 mg/kg) intraperitoneally once daily for 3 weeks (n=6 per group). **(A)** The mice weights were examined every tree days. **(B)** H&E staining in liver, lung, heart, kidney spleen. **(C,D)** HepG2, Huh-7, Hep3B and LO2 cells were treated with vehicle or indicated concentrations of Bkh126 for 48 h and subjected MTT assays (**C**). The IC50 index for the growth inhibition of each cell line were evaluated (**D**).
